# Reduced neural responsiveness to looming stimuli is associated with increased aggression

**DOI:** 10.1093/scan/nsab058

**Published:** 2021-05-07

**Authors:** R James Blair, R u Zhang, Johannah Bashford-Largo, Sahil Bajaj, Avantika Mathur, Jay Ringle, Amanda Schwartz, Jaimie Elowsky, Matthew Dobbertin, Karina S Blair, Patrick M Tyler

**Affiliations:** Center for Neurobehavioral Research, Boys Town National Research Hospital, Boys Town, NE 68154, USA; Center for Neurobehavioral Research, Boys Town National Research Hospital, Boys Town, NE 68154, USA; Center for Neurobehavioral Research, Boys Town National Research Hospital, Boys Town, NE 68154, USA; Center for Neurobehavioral Research, Boys Town National Research Hospital, Boys Town, NE 68154, USA; Center for Neurobehavioral Research, Boys Town National Research Hospital, Boys Town, NE 68154, USA; Translational Research Center, Boys Town, NE 68154, USA; Center for Neurobehavioral Research, Boys Town National Research Hospital, Boys Town, NE 68154, USA; Center for Neurobehavioral Research, Boys Town National Research Hospital, Boys Town, NE 68154, USA; Center for Neurobehavioral Research, Boys Town National Research Hospital, Boys Town, NE 68154, USA; Center for Neurobehavioral Research, Boys Town National Research Hospital, Boys Town, NE 68154, USA; Translational Research Center, Boys Town, NE 68154, USA

**Keywords:** aggression, looming stimuli, inferior frontal gyrus, amygdala

## Abstract

While neuro-cognitive work examining aggression has examined patients with conditions at increased risk for aggression or individuals self-reporting past aggression, little work has attempted to identify neuro-cognitive markers associated with *observed/recorded* aggression. The goal of the current study was to determine the extent to which aggression by youth in the first three months of residential care was associated with atypical responsiveness to threat stimuli. This functional MRI study involved 98 (68 male; mean age = 15.96 [sd = 1.52]) adolescents in residential care performing a looming threat task involving images of threatening and neutral human faces or animals that appeared to be either loom or recede. Level of aggression was negatively associated with responding to looming stimuli (irrespective of whether these were threatening or neutral) within regions including bilateral inferior frontal gyrus, right inferior parietal lobule, right superior/middle temporal gyrus and a region of right uncus proximal to the amygdala. These data indicate that aggression level is associated with a decrease in responsiveness to a basic threat cue-looming stimuli. Reduced threat responsiveness likely results in the individual being less able to represent the negative consequences that may result from engaging in aggression, thereby increasing the risk for aggressive episodes.

## Introduction

Aggressive and antisocial behaviors are a leading cause of all child and adolescent referrals to mental health clinicians (Coghill, 2013), and aggression in residential youth care institutions is a frequent problem (Barzman *et al.*, 2011; Cornaggia *et al.*, 2011). Aggression is associated with an exceptionally high societal and economic burden (Erskine *et al.*, 2014). As such, aggression is a serious social concern that incurs significant costs not only for victims and perpetrators but also for society more generally.

Many factors place the individual at increased risk for the commission of aggression (Blair, 2016; Mitjans *et al.*, 2019); e.g. economic deprivation (Farrington, 1989; Piotrowska *et al.*, 2019), poor child-rearing (Farrington, 1989; Piotrowska *et al.*, 2019), attention deficit hyperactivity disorder (ADHD; Farrington, 1989; Siever, 2008), psychopathy/callous-unemotional (CU) traits (Anderson and Kiehl, 2014; Robertson *et al.*, 2020), early life stressor exposure (ELS; Caspi *et al.*, 2002; Shonkoff and Garner, 2012) and past aggression (Farrington, 1989). Recent interest has focused on neurocognitive mechanisms that, when dysfunctional, are associated with aggression (Viding *et al.*, 2012; Blair *et al.*, 2018; Kiehl *et al.*, 2018). Work has, for example, considered neurocognitive systems that appear compromised in adolescents with conduct disorder (CD; Blair *et al.*, 2018; Fairchild *et al.*, 2019) or adults with psychopathy (Anderson and Kiehl, 2014).

A core neurocognitive system that, when dysfunctional, increases aggression risk is that mediating the response to threat (Blair *et al.*, 2018). Interestingly, both increased and decreased threat responsiveness dysfunction have been related to an increased aggression risk. With respect to increased threat responsiveness, this is thought to particularly increase the risk for irritability (Leibenluft, 2017; Crum *et al.*, 2020) and reactive aggression, i.e. aggression made in response to threat, social provocation or frustration (Dodge *et al.*, 1995; Lopez-Duran *et al.*, 2009). The individual responds with reactive aggression/rage rather than flight or freezing to provocation (Blair, 2004). In line with this view, adolescents and adults at increased risk for reactive aggression have been reported to show increased amygdala and periaqueductal gray responses and/or reduced responsiveness within ventromedial prefrontal cortex/orbitofrontal cortex to threat (Lee *et al.*, 2009; Choe *et al.*, 2015; White *et al.*, 2016).

In contrast, individuals less responsive to threat may be less likely to represent the negative valence of consequences of aggression (e.g. punishment and risking getting hurt by the intended victim). Moreover, the neural systems responding to threat also respond to the distress of other individuals (Blair *et al.*, 2018), as such the individual may care less about harming other individuals to achieve their goals (Blair *et al.*, 2015). In line with this, considerable functional magnetic resonance imaging (fMRI) data reveal that youth with conduct problems and psychopathic or CU traits (i.e. those with reduced empathy who are at increased risk for instrumental aggression) show reduced amygdala responses to distress cues (Marsh *et al.*, 2008; Jones *et al.*, 2009; Passamonti *et al.*, 2010; Lozier *et al.*, 2014) and threat stimuli (Sterzer *et al.*, 2005; Hwang *et al.*, 2016). Indeed, using a version of the task used in this study, we found that adolescents with disruptive behavior disorders showed decreased activity to looming (particularly looming threat) stimuli within lateral frontal, parietal, insula and temporal cortices and uncus (White *et al.*, 2018).

Most of the previous work examining the neurocognitive underpinnings of aggression has investigated populations that met criteria for psychopathy or psychiatric diagnoses associated with aggression (e.g. CD or antisocial personality disorder) or self-reported aggression (Espinoza *et al.*, 2018; Hwang *et al.*, 2018; Sethi *et al.*, 2018). Little work has been attempted to identify neurocognitive markers associated with observed/recorded aggressive episodes. The goal of the current study was to determine the extent to which aggression by youth in residential care was associated with atypical responsiveness to threat stimuli.

Participants were presented with an adapted version of the looming task (Coker-Appiah *et al.*, 2013) previously used with an independent sample of youth with CD (White *et al.*, 2018). In this task, participants respond to threats or neutral stimuli that appear to either loom toward or recede away from them. Looming stimuli are prototypical elicitors of acute threat system activity in animal (Blanchard *et al.*, 1977) and human fMRI studies (Mobbs *et al.*, 2007; Coker-Appiah *et al.*, 2013). We made two contrasting hypotheses. First, and suggestive of the association between increased threat responsiveness and reactive aggression (cf. Lee *et al.*, 2009; Choe *et al.*, 2015; White *et al.*, 2016), we predicted that increased aggression within residential care would be associated with increased activity to threat stimuli (looming/threat stimuli) within neural systems mediating the response to threat (i.e. lateral frontal, parietal, insula and temporal cortices, the amygdala and periaqueductal gray). Second, and following previous findings on this task with adolescents with disruptive behavioral disorders (White *et al.*, 2018), we predicted that increased aggression within residential care would be associated with decreased activity to looming (looming/threat stimuli) stimuli within the neural systems mediating the response to threat. These predicted associations would manifest as regions showing significant aggression level-by-direction or aggression level-by-emotion interactions.

## Methods

### Participants

Study participants were 98 youths from a residential care facility (68 men; average age = 15.96 years). An additional five participants were scanned but excluded due to excessive movement (>10% volumes censored at >0.5 mm motion across adjacent volumes, *n *= 3), low accuracy on the task (<80% response rate, *n *= 1) or scanning/processing errors (*n *= 1). Clinical characterization was completed through psychiatric interviews by licensed psychiatrists with the participant and a parent/legal guardian following standard clinical practice.

The exclusion criteria for participants in the study included pervasive developmental disorder, Tourette’s syndrome, lifetime history of psychosis, neurological disorder, head trauma, non-psychiatric medical illnesses requiring medications that may have psychotropic effects (e.g. beta-blockers, steroids) and Intelligence Quotient (IQ) <75. Institutional review board approval was acquired before data collection began. Informed consent was obtained from a parent/legal guardian, and informed assent was obtained from the youth.

### Measures

#### Aggression level: behavioral incident reports.

Significant behavioral incident report data were collected from the facility’s electronic youth records based on daily staff observations that were documented and reported to a program supervisor within 24 h. Reports included the date, time and description of the event and were coded according to established definitions based on the incident type. Incident types included verbal aggression, physical aggression, property destruction, assault on youth and assault on adults. Definitions for the incidents were acquired from the training manual for the program. For example, physical aggression was defined as ‘Program participant engages in physically aggressive behaviors such as throwing objects, slamming doors, overturning furniture, or slamming fists.’ The participants’ aggression level was their total number of aggressive behavioral incidents for the first 3 months of the youths’ placement was calculated. In the absence of context details, the reports of the aggressive episodes could not be classified as instrumental or reactive.

#### Psychiatric symptom severity and ELS assessments.

Psychopathology was indexed via youth self-report and parent report on the following measures: (i) The Reactive-Proactive Questionnaire (RPQ; Raine *et al.*, 2006), a validated measure of both proactive and reactive aggression in youth (Cima *et al.*, 2013); (ii) the Inventory of Callous-Unemotional Traits (ICU; Frick, 2004), a measure of callous-unemotional traits (CU); (iii) the Affective Reactivity Index (ARI; Stringaris *et al.*, 2012), a measure of irritability over the past 6 months and (iv) the Mood and Feelings Questionnaire (Angold *et al.*, 1995), a measure of depression symptomatology. In addition, exposure to ELS was indexed by the Childhood Trauma Questionnaire (CTQ; Bernstein *et al.*, 1997).

#### Looming task.

The participants performed a looming task (adapted from Coker-Appiah *et al.*, 2013); see Supplementary Figure S1. They were presented with an image that appeared to either loom toward or recede away from them. Images were human or animal faces and were either threatening or neutral, i.e. there were eight different trial types—2 (Loom *vs* Recede) × 2 (Human *vs* Animal) × 2 (Threat *vs* Neutral). Images were presented rapidly in a series of 16 50-ms frames of increasing or decreasing size in the center of the screen to create the effect of either looming (i.e. increasing in size in rapid succession) or receding (i.e. decreasing in size in rapid succession; total stimulus duration: 800 ms). Stimulus presentations were followed by a fixation point, which was on screen for a jittered duration of 1250–4250 ms. The task included one block of 160 stimuli (20 of each of the eight trial types). Image order was randomized across participants. In order to ensure attention to the task, participants were instructed to press a button with their right index finger as quickly as possible when an image appeared on the screen.

#### MRI parameters.

All data were collected on a 3 T Siemens Skyra scanner. A total of 197 functional images were taken with a T2* weighted gradient echo planar imaging (EPI) sequence (repetition time = 2500 ms; echo time = 27 ms; 240 mm field of view; 94 × 94 matrix; 90^o^ flip angle). Whole-brain coverage was obtained with 43 axial slices (thickness, 2.5 mm; voxel size 2.6 × 2.6 × 2.5 mm^3^). A high-resolution T1 anatomical scan (MP-RAGE, repetition time = 2200 ms; echo time = 2.48 ms; 230 mm field of view; 8^o^ flip angle; 256 × 208 matrix; thickness, 1 mm; voxel size 0.9 × 0.9 × 1 mm^3^) in register with the EPI dataset was obtained covering the whole brain with 176 axial slices.

#### Functional MRI analysis: data preprocessing and individual-level analysis.

fMRI data were preprocessed and analyzed using Analysis of Functional NeuroImages (AFNI) software (Cox, 1996). Both individual- and group-level analyses were conducted. At the individual level, functional images from the first four repetitions, collected prior to equilibrium magnetization, were discarded. The participants’ anatomical scans were then individually registered to the Talairach and Tournoux atlas (Talairach and Tournoux, 1988). The individuals’ functional EPI data were then registered to their Talairach anatomical scan. The EPI datasets for each participant were spatially smoothed (isotropic 6 mm^3^ Gaussian kernel) to reduce variability among individuals and generate group maps. Next, the time series data were normalized by dividing the signal intensity of a voxel at each time point by the mean signal intensity of that voxel for each run and multiplying the result by 100, producing regression coefficients representing percent-signal change. Every TR on which motion exceeded 1 mm was censored.

Eight regressors were generated: Looming Animal Negative, Looming Animal Neutral, Looming Human Negative, Looming Human Neutral, Receding Animal Negative, Receding Animal Neutral, Receding Human Negative and Receding Human Neutral. GLM fitting was performed with these eight regressors, six motion regressors, and a regressor modeling baseline drift. All regressors were convolved with a canonical hemodynamic response function (HRF) to account for the slow hemodynamic response (with time point commencing at time of the first image onset). This produced a β coefficient and associated *t* statistic for each voxel and regressor. There was no significant regressor collinearity.

### Statistical analyses

To reduce the possibility of outlier scores having a disproportionate impact on the data, Rankit transformations were applied to participants’ aggression level (pretransformation skewness and kurtosis scores: 2.98 & 10.37; post scores: 0.81 & −0.32). The aggression level was then *z*-scored and these values were used as continuous covariates in analyses.

#### Clinical correlations.

Correlation analyses were conducted to determine the associations between Rankit transformed aggression scores, age, IQ, sex, psychiatric diagnostic status, RPQ, ICU, ARI, MFQ and ELS as indexed by the CTQ and current medication status. A follow-up multiple regression analysis was conducted to determine the extent to which reported/observed aggression could be predicted on the basis of these clinical and demographic variables.

#### Behavioral data.

A 2 (Direction: looming, receding) × 2 (Stimulus: animal, human) × 2 (Emotion: negative, neutral) repeated measures Analysis of Covariance (ANCOVA) was conducted on the reaction time data with Rankit-transformed and z-scored aggression-level scores and IQ scores as the covariates.

#### MRI data.

To examine relationships between aggression levels and neural responses to looming threats, a full 2 (Direction: looming, receding) × 2 (Stimulus: animal, human) × 2 (Emotion: negative, neutral) repeated measures ANCOVA was conducted on the BOLD response data via 3dMVM. Rankit-transformed and *z*-scored aggression scores were used as continuous covariates. Correction for multiple comparisons was performed using a spatial clustering operation in AFNI’s 3dClustSim utilizing the autocorrelation function with 10 000 Monte Carlo simulations for a whole-brain gray matter mask with an empirical blur of 9.889. The initial threshold was set at *P* = 0.001 (Cox *et al.*, 2017a,b). This procedure yielded an extant threshold of *k *= 19 voxels, which then results in a cluster-level false-positive probability of *P* < 0.05, corrected for multiple comparisons. To facilitate future meta-analytic work, effect sizes [partial η^2^ (pη^2^)] for all clusters are reported. Interactions of covariates with variables identified via the ANCOVAs were interpreted via correlational analyses using SPSS 22.0 (*P* < 0.05). Core interactions with respect to our hypotheses were: aggression level-by-direction and aggression level-by-direction-by-emotion. All SPSS follow-up analyses (including the correlational analyses mentioned below) were conducted on BOLD response data extracted from the clusters showing significant aggression level-by-direction interactions.

#### Associating functional signal with symptom profiles.

Correlational analyses were conducted to determine the extent of differential responses with respect to our core hypothesized interactions (aggression level-by-direction and aggression level-bydirection-by-emotion) and clinical variables: RPQ, ICU, ARI, MFQ and CTQ scores and CD or ADHD diagnostic status.

#### Potential confounds.

Given that a number of participants received diagnoses of CD (*N* = 62) and ADHD (*N* = 69), our main ANCOVA was repeated twice with presence/absence of CD and ADHD as group variables. In addition, to explore the association of BOLD response with raw aggression level scores, the ANCOVA was repeated with raw, rather than Rankit transformed, aggression level scores as the covariate.

## Results

### Demographics and clinical correlations

Table 1 reports the demographic variables, incidence rates of psychiatric conditions, symptom levels and medication rates for the samples and correlations of these variables with number of aggression incidences. All correlations were non-significant except those with IQ and parent-reported preadmission aggression (i.e. RPQ and RPRS scores). Aggression level was strongly positively associated with self-reports of both precare reactive and instrumental aggression.

**Table 1.** Demographic and clinical variables

	Mean	s.d.	*r*	*P*	*B*	*P*
Age	15.96	1.52	−0.07	0.49	−0.08	0.40
IQ	100.42	13.06	**−0.23**	0.03	−0.07	0.49
Sex	68 (male)	69.40%	0.13	0.22	0.11	0.25
CD	62	63.30%	0.06	0.56	−0.13	0.15
ADHD	69	70.40%	0.03	0.76	−0.06	0.56
GAD	31	31.60%	−0.14	0.18	−0.17	0.08
MDD	31	31.60%	−0.17	0.1	−0.10	0.34
RPQ total score	11.73	8.53	**0.44**	0	**0.62**	0.00
RPQ proactive	2.91	3.84	**0.41**	0		
RPQ reactive	8.82	5.42	**0.4**	0		
ARI	3.29	3.31	0.14	0.17	−0.16	0.22
ICU	23.93	7.89	−0.03	0.78	**0.22**	0.03
MFQ	12.66	12.77	−0.1	0.31	**−0.24**	0.01
Total CTQ	40.89	14.73	−0.1	0.32	−0.14	0.20
EA	9.73	4.65	−0.04	0.67		
PA	7.61	3.68	−0.02	0.87		
SA	6.35	3.79	−0.05	0.64		
EN	9.45	4.45	−0.15	0.15		
PN	7.74	3.85	−0.1	0.32		
Antipsychotic	7	7.10%	0.16	0.13	0.10	0.28
Stimulant	20	20.40%	0.13	0.2	0.11	0.24
SSRI	19	19.40%	−0.03	0.74	0.02	0.87

s.d. = standard deviation; *r*s are correlations with aggression level; *r*s for categorical variables scored as 1: male, 0: female, 1: diagnosis, 0: not present, 1: medication, 0: no medication; *B*s and associated *P*s for the multiple regression predicting observed/reported aggression severity using the variables marked; ADHD = attention deficit/hyperactivity disorder, CD = conduct disorder, MDD = major depressive disorder, GAD = generalized anxiety disorder, ARI = Affective Reactivity Index, ICU = Inventory of Callous Unemotional traits; MFQ = Mood and Feelings Questionnaire; CTQ = Child Trauma Questionnaire; EA = emotional abuse; PA = physical abuse; SA = sexual abuse; EN = emotional neglect; PN = physical neglect. Significant correlations are bolded.

The multiple regression for observed/reported aggression severity including all the variables in Table 1 (total RPQ and CTQ scores but not the subscales of these scales) revealed a significant regression equation [F(3,86) = 12.72; *P* < 0.001]. R^2^ was 0.31. Significant predictors for observed/reported aggression were total RPQ score (standardized B = 0.62; *P* < 0.001), MFQ (standardized B = −0.24; *P* = 0.014) and ICU (standardized B = 0.22; *P* = 0.028). All other variables were not significant predictors.

### Behavioral data on the task

This analysis revealed a highly significant main effect of direction (F1,91) = 85.12; *P* < 0.001; pη^2^ = 0.48); participants were slower to respond to looming stimuli than receding stimuli (mean_loom_ = 468.14 [s.e. = 14.16]; mean_recede_ = 419.54 [s.e. = 10.25]). However, there were no other significant main effects or interactions.

### Movement data

Volumes were censored if there was >0.5 mm motion across adjacent volumes. Within the 98 participants sample, no participant had >5% censored volumes. Importantly, there were no significant correlations between aggression level and censored volumes, average motion per volume and maximum displacement during scanning within the final sample [*r*s = −0.161 to 0.01; *ns*].

### fMRI data

Our initial analysis revealed regions showing interactions relevant to our hypotheses: aggression level-by-direction and aggression level-by-direction-by-emotion (although no regions showed significant aggression level-by-emotion interactions). Regions showing significant main effects of direction, emotion and type as well as direction-by-emotion and direction-by-type are reported in the Supplemental Material (Supplementary Table S1).

#### Aggression level-by-direction.

**Fig. 1.**
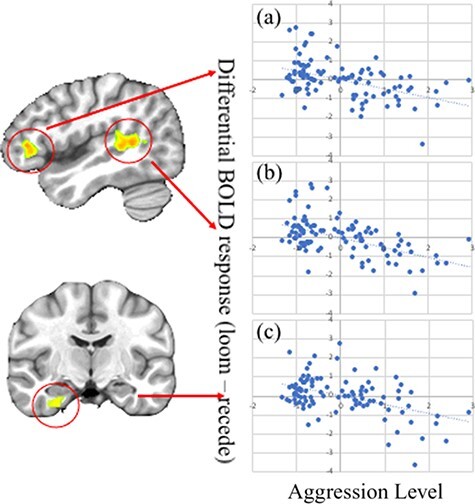
Aggression level-by-direction interaction within: (a) right IFG; (b) right STG/MTG; and (c) right uncus. Scatterplots depict the partial correlations and adjusted residuals for each of the regions. Adjusted residuals for the Rankit transformed *z*-scored aggression levels are plotted against adjusted residuals for the average differential bold responses to looming relative to receding trials.

Significant aggression level-by-direction interactions were observed within regions including bilateral inferior frontal gyrus (IFG), right inferior parietal lobule (IPL), right superior temporal and middle temporal gyrus (STG)/(MTG) and right uncus; see Figure 1 and Table 2. Within all these regions, aggression level was significantly negatively associated with differential responsiveness to looming relative to receding stimuli (across regions: range of partial *rs* = −0.42 to −0.53, *P* < 0.001); see Figure 1. Notably, this reflected significant negative associations in all regions between aggression level and responsiveness to looming stimuli (r = −0.22 to −0.33, *P* = 0.03 to 0.001—although for right IFG, this was a trend; r = −0.19, *P* = 0.063). Aggression level showed no significant relationship within any of these regions to receding stimuli (r = 0.003 to 0.157, *P* = 0.977 to 0.124).

**Table 2.** Significant areas of activation from the ANCOVA analysis

Region	BA	Voxels	*X*	*Y*	*Z*	*F*-value	pη^2^
Aggression level-by-direction							
R. IFG	46/47	44	44	32	5	25.46	0.211
L. IFG	13/45/47	23	−43	23	5	19.792	0.172
R. MTG/STG	41	104	44	−43	8	36.23	0.276
R. IPL	40	37	29	−40	56	21.503	0.185
R. Uncus	20	27	29	−13	−28	26.859	0.22
L. Culmen		39	−19	−46	−19	30.227	0.241
Aggression level-by-direction-by-emotion							
R. Precuneus/PCC	7	67	2	−58	32	20.554	0.178

*Note*: Activations are from whole brain analyses significant at *P* < 0.001, corrected for multiple comparisons (significant at *P* < 0.05).

IFG: inferior frontal gyrus; MTG: middle temporal gyrus; STG: superior temporal gyrus; IPL: inferior parietal lobule; PCC: posterior cingulate cortex.

#### Aggression level-by-direction-by-emotion.

There was a significant aggression level-by-direction-by-emotion interaction within precuneus/posterior cingulate cortex (PCC). Within this region, aggression level was significantly negatively associated with responsiveness to looming threat (*r *= −0.31, *P *= 0.002) but not to looming neutral, receding threat or receding neutral (*r *= −0.014,0.132,−0.097, *P *= 0.892,0.197,0.345, respectively).

#### Correlation analyses associating functional signal with symptom profiles.

Differential responses to looming versus receding stimuli within three of the six regions identified through the aggression level-by-direction interaction (MTG/STG, right IFG and IPL), and the region of precuneus showing the reduced responsiveness to looming threats via the aggression level-by-direction-by-emotion interaction, all showed significant negative associations with prior levels of aggression as indexed by the RPQ (r = −0.385,−0.33,−0.242 & −0.284 *P* < 0.001, = 0.001, = 0.02 & = 0.006, respectively). Moreover, differential responses to looming *vs* receding stimuli within two additional regions identified through the aggression level-by-direction interaction (uncus and left IFG) showed trend negative associations with prior levels of aggression as indexed by the RPQ (r = −0.177,−0.188; *P* = 0.091, = 0.073, respectively). In contrast, differential responses with none of these regions related to ICU or ARI, diagnoses of ADHD or CD or MFQ scores (with one exception—differential responses to looming *vs* receding stimuli within MTG/STG were negatively associated with MFQ scores; r = −0.201, *P* = 0.049).

#### Potential confounds.

To rule out the possibility that the results simply represent the manifestation of an ADHD or CD diagnosis, our main analysis was repeated twice—once with ADHD diagnosis/not as a group variable and once with CD diagnosis/not as a group variable. The results of these analyses closely replicated the results of our main analysis (Supplementary Tables S1 and S2). Similarly, exploring the association of bold responses with raw aggression-level scores also largely replicated the results of our main analysis with the exception of bilateral IFG (Supplementary Table S3).

## Discussion

The goal of the current study was to determine the extent to which aggression by youth in the first 3 months of residential care was associated with atypical responsiveness to threat stimuli—specifically whether it was associated with increased or decreased responsiveness to threat. These hypotheses were generated from previous data on individuals with conduct problems and differential relationships with reactive and instrumental aggression. The current data clearly indicated that aggression level in this sample was associated with reduced threat responsiveness within regions including inferior frontal, parietal and temporal cortices.

Before considering the BOLD response data it is worth briefly considering the multiple regression results. In line with the data indicating the importance of past aggression predicting current and future aggression (Farrington, 1989), observed/recorded aggression within residential care was particularly predicted by past aggression (RPQ) as well as CU scores and depression (MFQ) severity. Variables that might have been expected to predict aggression such as ADHD diagnostic status (cf. Farrington, 1989; Siever, 2008) and ELS exposure (cf. Caspi *et al.*, 2002; Shonkoff and Garner, 2012) were not significant predictors in this regression. However, they were significant predictors of RPQ scores (see Supplemental Material 1). The slight inconsistency between these regression results might reflect the greater period of record relating to preadmission RPQ scores relative to the time in residential care. It is also possible that removal from the home context associated with ELS could have reduced the association between this variable and aggression. The individual would be less exposed to threat, less threat-sensitive and thus less likely to react to provocation (threat, social provocation or frustration) with reactive aggression. Importantly, though, the strong association between observed/recorded aggression within residential care and self-reports of aggression precare suggests a commonality in individual risk even within these different environmental circumstances.

With respect to the BOLD response data, this study indicated that level of aggression was strongly associated with reduced threat responsiveness to looming *vs* receding stimuli—though level of aggression was not associated with reduced responsiveness to threatening versus neutral images. The lack of an association with threatening versus neutral images may reflect that in this task, at least, the direction manipulation is a far stronger elicitor of threat-related activity than the emotion manipulation. This can be seen in Supplementary Table S1 where the direction manipulation was associated with responsiveness in a wider number of regions than the emotion manipulation, and that while the direction manipulation resulted in bilateral amygdala responses, the emotion main effect was not associated with amygdala activity.

Previously, reduced threat responsiveness has been reported in clinically aggressive individuals who present with high levels of instrumental and reactive aggression as opposed to high levels of reactive aggression alone (Hwang *et al.*, 2016; White *et al.*, 2018). Notably, level of aggression in the current participants was associated with prior levels of both past instrumental and reactive aggression on the RPQ. It is argued that responsiveness to threat is likely inversely associated with aggression level for two main reasons. First, individuals less responsive to threat may be less likely to represent the negative valence of the negative future consequences that may result from engaging in aggression (e.g. punishment). Moreover, the neural systems responding to threat also respond to the distress of other individuals (Blair *et al.*, 2018). Reduced responsiveness to the distress of others means that the individual cares less about harming other individuals to achieve their goals, and thus may be more likely to engage in aggression to achieve these goals (Blair *et al.*, 2015).

The neural regions that showed differential responsiveness as a function of aggression level included regions implicated in emotional attention (i.e. IFG, IPL and MTG/STG) (Pourtois *et al.*, 2013) as well as uncus. It is worth noting that reduced responsiveness within regions implicated in attention to salient stimuli, including emotional stimuli, was seen in an independent sample of adolescents with disruptive behavior disorders (DBDs) relative to comparison adolescents (White *et al.*, 2018); i.e. IFG, MTG and uncus. Moreover, within the youths with DBDs, CU traits were inversely related to IFG, IPL and STG (White *et al.*, 2018). Notably, in the current study, responsiveness within most of the regions showing reduced threat responsiveness as a function of participants’ levels of aggression were also negatively related to self-reported historical aggression levels (RPQ scores)—although not level of CU traits.

Given the focus on emotional attention, it might have been expected that we would also see a significant negative association between aggressive episodes and amygdala responsiveness to looming relative to receding stimuli. The amygdala has long been considered critically involved in emotional processing (Phelps and LeDoux, 2005; LeDoux, 2007) and emotional attention (Vuilleumier, 2005), priming neurons within temporal cortex involved in the emotional stimulus as well as neurons coding spatial information within parietal cortex potentially via PCC (Vuilleumier, 2005; Luo *et al.*, 2007). However, no significant aggression level by direction interaction was seen within the amygdala in the current study at stringent statistical thresholds—although there were activations within relatively proximal regions of uncus/parahippocampal gyrus (and these extended into the amygdala at more relaxed statistical thresholds). There was a highly significant main effects of direction (loom > recede) within the amygdala that extended into parahippocampal gyrus/uncus (see Supplementary Figure S2). While uncus/the parahippocampal gyri have been implicated in emotion-based judgments (Begue *et al.*, 2019), they are not typically considered critical for generating the emotional response (e.g. Tye, 2018). As such, it can thus be speculated that the region of parahippocampal gyrus showing reduced responding to looming stimuli as a function of aggression level in the adolescents, at least partially reflects reduced amygdala activity. Supporting this speculation, an exploration of responsiveness within the region of amygdala showing the main effect of direction to looming stimuli revealed that loom *vs* recede differential BOLD responsiveness was significantly negatively associated with aggression level (see Supplemental Analysis 2 and Supplementary Figure S2).

Two caveats should be considered. It could be argued that these results simply reflected the psychiatric status of the participants, that they are manifestations of the participants’ diagnoses of ADHD and/or CD. Both ADHD and CD are associated with significantly increased risks for aggression (Blair *et al.*, 2014; Mogavero *et al.*, 2018; Fairchild *et al.*, 2019), and a significant proportion of the participants in the current study had ADHD and/or CD diagnoses (70% and 63%, respectively; see Table 1). However, it is important to note that neither diagnostic status of ADHD nor even CD was significantly associated with aggression level during this time period. Moreover, inclusion of group variables (ADHD or CD) into the ANCOVA in follow-up analyses did not alter the significant findings observed in our main analysis (see Supplemental Material 2; Supplementary Tables S1 and S2). In addition, and notably with respect to ADHD, it is worth noting that ADHD is more typically comorbid with anxiety—rather than being protective of anxiety (Felt *et al.*, 2014). This is more consistent with an association between ADHD and increased threat responsiveness (Brotman *et al.*, 2010; Van Dessel *et al.*, 2018).

The situation is more complex with respect to CD. Aggression is obviously a core feature of CD. Moreover, previous work with this task has reported that patients with DBD (including CD) show reduced threat responsiveness on the looming task in proximal regions (White *et al.*, 2017). We assume that the form of pathophysiology seen here (reduced neural threat responsiveness) is a feature of a significant number of participants with CD. However, there is heterogeneity within cases with CD (Blair *et al.*, 2015). Not all patients with CD show reduced threat responsiveness; some indeed show increased threat responsiveness (Blair *et al.*, 2015; Fairchild *et al.*, 2019). In short, we believe that the current findings, revealing an association between decreased threat responsiveness and aggression, reflect this core form of pathophysiology that increases the risk for aggression. We believe that the lack of relationship of this pathophysiology (and indeed aggression level) with CD diagnosis reflects (i) the high incidence rate of CD in this sample (63%); (ii) the potential reduced power of a dichotomous variable relative to the dimensional aggression-level variable and (iii) that a significant number of cases of CD are associated with increased threat responsiveness. While increased threat responsiveness may increase the risk for aggression in threatening/frustrating environments, this risk is mitigated in the residential treatment environment.

The second caveat relates to the recent concerns that have been raised with respect to individual difference research generally given the poor test–retest reliability results of BOLD response and, for that matter, resting state and behavioral data (Hedge *et al.*, 2017; Noble *et al.*, 2019; Elliott *et al.*, 2020). This is undeniably a concern. However, with respect to the current results, it is important to note that (i) the number of trials per condition related to the individual difference finding was relatively large. We observed a direction by aggression interaction. There were 80 looming and 80 receding trials per individual that went into this contrast and (ii) the number of participants was also relatively large (*N = *98). Increases in the number of trials within a paradigm and the number of participants who are assessed on the paradigm increase the confidence we can have with respect to individual difference data (Hedge *et al.*, 2017; Haines *et al.*, 2020). However, these concerns are highly salient when considering how the current paradigm or comparable neurocognitive behavioral measures could be translated into clinical utility. New analytical techniques for behavioral data at least offer reasons for optimism for the future (Haines *et al.*, 2020).

In conclusion, this is one of the first studies to examine the neural correlates associated with an increased risk for observed aggressive episodes in a specific context rather than self-reports of aggression or a specific diagnostic category. Consistent with previous work, adolescents’ level of aggression was negatively associated with responsivity to looming stimuli in regions implicated in emotional attention and responding; i.e. uncus/hippocampal gyrus/amygdala. The current data have several clinical implications. Supporting previous work, the current data suggest that risk for aggression is strongly related to past aggression levels (Farrington, 1989). Further, they suggest that individuals who have shown past aggression and who are also less threat-responsive are at particular risk. Some information on emotional responsiveness might be gleaned from self-reported anxiety, depression and CU symptomatology. However, previous data (consistent with the relatively weak predictive power of these variables in the current data) indicate that there are complex interrelationships between these self-reported symptoms and underlying pathophysiology (Kimonis *et al.*, 2012; Meffert *et al.*, 2018). As such, more direct measures of underlying pathophysiology may become important. Moreover, such measures would be important for tracking treatment efficacy. Indeed, it will be important to determine the extent to which treatment-induced normalization of this reduced threat responsiveness is associated with reduction in risk for future aggression.

## Supplementary Material

nsab058_Supp
