# Supplementary material for: Reduced neural responsiveness to looming stimuli is associated with increased aggression
Source: Soc Cogn Affect Neurosci. 2021 May 7;16(10):1091–9. doi: 10.1093/scan/nsab058 (PMC8483278; doi:10.1093/scan/nsab058)
Supplement: nsab058_Supp [file nsab058_supp.zip › Supplemental_Material_Loom_Agg-kb.docx]

**Supplemental Material 1: Additional analysis:**

*Multiple Regression (total RPQ score):* The multiple regression for total RPQ score included all non-RPQ variables in Table 1 and also revealed a significant regression equation [F(5,84)=34.47; P<0.001]. R^2^ was 0.67.  Significant predictors for total RPQ score were ARI (standardized B=0.48; p<0.001), observed/reported aggression (standardized B=0.38; p<0.001), ICU (standardized B=0.23; p=0.002) total CTQ score (standardized B=16; p=0.019) and ADHD diagnostic status (standardized B=0.14; p=0.029). All other variables were not significant.

**Supplemental Material 2: Amygdala responsiveness:**

***Introduction:*** Our main analysis revealed no significant Aggression Level-by-Direction interaction within the amygdala in the current study at stringent statistical thresholds – though there were activations within relatively proximal regions of uncus/parahippocampal gyrus (and these extended into the amygdala at more relaxed statistical thresholds). There was a highly significant main effects of Direction (Loom>Recede) within the amygdala that extended into parahippocampal gyrus/uncus (see Supplemental Figure 2). While uncus/the parahippocampal gyri have been implicated in emotion-based judgements (Begue et al., 2019), they are not typically considered critical for generating the emotional response (e.g., Tye, 2018). As such, it can thus be speculated that the region of parahippocampal gyrus showing reduced responding to looming stimuli as a function of aggression level in the adolescents, at least partially reflects reduced *amygdala* activity. To explore this issue further, we examined amygdala responsiveness within the amygdala regions showing significantly greater responses to Looming vs. Receding stimuli in the main effect of Direction (see Supplemental Table 1).

Method: BOLD responses to Looming and Receding stimuli within the region of left amygdala showing a main effect of Direction (see Supplemental Figure 2a). The Looming vs. Receding difference was then correlated with Aggression level within residential care as well as RPQ total and both proactive and reactive subscale scores.

Results: Correlation analyses revealed significant negative associations between Looming vs. Receding BOLD differential response and Aggression level within residential care (r=-0.211; p=0.037), RPQ total score (r=-0.237; p=0.023) and proactive RPQ subscale score (r=-0.276; p=0.008); see Supplemental Figure 2. There was no significant association between Looming vs. Receding BOLD differential response and reactive RPQ subscale score.

**Supplemental Material 3: Additional tables:**

*Supplemental Table 1.* Significant areas of activation from the ANCOVA analysis. Activations are from whole brain analyses significant at p<0.001, corrected for multiple comparisons (significant at p<0.05).

| Region | BA | Voxels | X | Y | Z |
| --- | --- | --- | --- | --- | --- |
| Direction | | | | | |
| R Lateral Frontal Gyrus | 9 | 37 | 41 | 17 | 29 |
| R. Anterior Insula | 13 | 50 | 35 | 14 | 14 |
| L. Inferior Parietal Lobule | 40 | 33 | -49 | -55 | 44 |
| L. Posterior Insula | 13 | 82 | -52 | -31 | 20 |
| R. Precuneus | 7 | 129 | 8 | -67 | 38 |
| L. Culmen |  | 13 | -19 | -46 | -19 |
| R. MTG | 22 | 447 | 62 | -31 | 2 |
| L. STG | 41 | 23 | -58 | -19 | 8 |
| R. Amygdala |  | 40 | 20 | -4 | -13 |
| L. Amygdala |  | 41 | -19 | -4 | -10 |
| R. Uncus | 28 | 20 | 26 | 2 | -28 |
| R. Thalmus extending to PAG |  | 55 | 20 | -28 | 2 |
| L. Thalamus |  | 51 | -19 | -25 | -1 |
| L. Lentiform Nucleus and Putamen |  | 28 | -22 | 8 | -4 |
| R/L. Lingual Gyrus | 18 | 4627 | -7 | -73 | -13 |
| R. Inferior Occipital Gyrus | 18 | 30 | 26 | -91 | -10 |
| L. Cerebellar Tonsil |  | 24 | -1 | -52 | -34 |
| Type | | | | | |
| R/L. VMPFC | 10 | 298 | 5 | 44 | -7 |
| R/L. RMPFC | 9 | 79 | 5 | 50 | 35 |
| R. IFG | 47 | 47 | 32 | 23 | -22 |
| R. PCC | 30 | 385 | 2 | -58 | 26 |
| L Inferior Parietal Lobule | 40 | 23 | -52 | -34 | 47 |
| R. STG | 39 | 304 | 47 | -58 | 20 |
| L. STG | 38 | 56 | -31 | 11 | -19 |
| R. MTG | 21 | 45 | 50 | -10 | -10 |
| L. MTG | 21 | 72 | -52 | -4 | -10 |
| L. MTG and STG | 39 | 68 | -40 | -58 | 23 |
| R. Amygdala |  | 48 | 20 | -7 | -13 |
| L. Amygdala |  | 33 | -16 | -7 | -10 |
| R/L. Lingual, Occipital, Fusiform Gyri | 18/36/37 | 6416 | -28 | -31 | -19 |
| L. Thalamus |  | 24 | -19 | -25 | 2 |
| Emotion | | | | | |
| R. STG | 42 | 45 | 62 | -22 | 8 |
| L. Cuneus | 18 | 31 | -7 | -73 | 20 |
| R. Lingual, Occipital, Fusiform Gyri | 18/36/37 | 511 | 32 | -85 | -7 |
| L. Lingual, Occipital, Fusiform Gyri | 18/36/37 | 709 | -31 | -85 | -7 |
| Direction-by-Type | | | | | |
| L. Cuneus | 17 | 24 | -7 | -82 | 8 |
| Direction-by-Emotion | | | | | |
| R. Lateral frontal gyrus | 9 | 30 | 35 | 11 | 41 |
| L. Superior parietal lobule | 7 | -36 | 31 | -58 | 41 |
| L. Precuneus | 7 | 25 | -7 | -55 | 62 |
| Type x Emotion |  |  |  |  |  |
| R. Cuneus |  | 117 | 2 | -70 | 20 |
| R. Middle Occipital Gyrus |  | 88 | 29 | -88 | -1 |
| R. Middle Occipital Gyrus |  | 33 | 47 | -70 | 8 |
| L. Inferior Occipital Gyrus |  | 35 | -28 | -85 | -7 |
| L. Lingual Gyrus |  | 25 | -19 | -52 | -1 |

Key to Supplemental Table 1: VMPFC: Ventromedial prefrontal cortex; RMPFC: Rostromedial prefrontal cortex; IFG: Inferior frontal gyrus; MTG: Middle temporal gyrus; STG: Superior temporal gyrus; IPL: Inferior parietal lobule; PCC: Posterior cingulate cortex; PAG: Periaquectal Gray.

*Supplemental Table 2.* Significant areas of activation from the ANCOVA analysis including ADHD as a group variable. Activations are from whole brain analyses significant at p<0.001, corrected for multiple comparisons (significant at p<0.05).

| Region | BA | Voxels | X | Y | Z |
| --- | --- | --- | --- | --- | --- |
| Aggression level-by-Direction | | | | | |
| R. IFG | 45/47 | 73 | 53 | 29 | 5 |
| L. IFG | 13/45/47 | 58 | -43 | 23 | 5 |
| R. MTG/STG | 42 | 86 | 53 | -31 | 17 |
| R. IPL | 40 | 7 | 29 | -40 | 56 |
| R. Uncus | 20 | 8 | 36 | -13 | -22 |
| L. Culmen |  | 13 | -19 | -46 | -19 |
| Aggression level-by-Direction-by-Emotion | | | | | |
| R. Precuneus/PCC | 7 | 8 | 2 | -58 | 32 |

Key to Supplemental Table 2: IFG: Inferior frontal gyrus; MTG: Middle temporal gyrus; STG: Superior temporal gyrus; IPL: Inferior parietal lobule; PCC: Posterior cingulate cortex.

*Supplemental Table 3.* Significant areas of activation from the ANCOVA analysis including CD as a group variable. Activations are from whole brain analyses significant at p<0.001, corrected for multiple comparisons (significant at p<0.05).

| Region | BA | Voxels | X | Y | Z |
| --- | --- | --- | --- | --- | --- |
| Aggression Level-by-Direction | | | | | |
| R. IFG | 45/47 | 65 | 47 | 29 | -1 |
| L. IFG | 45/46/47 | 61 | -46 | 29 | 8 |
| R. MTG/STG | 41 | 57 | 41 | -43 | 8 |
| R. IPL | 40 | 43 | 29 | -40 | 56 |
| R. Uncus | 20 | 18 | 29 | -10 | -28 |
| L. Culmen |  | 18 | -16 | -49 | -19 |
| Aggression Level-by-Direction-by-Emotion | | | | | |
| R. Precuneus/PCC | 7 | 39 | 2 | -58 | 35 |

Key to Supplemental Table 3: IFG: Inferior frontal gyrus; MTG: Middle temporal gyrus; STG: Superior temporal gyrus; IPL: Inferior parietal lobule; PCC: Posterior cingulate cortex.

*Supplemental Table 4.* Significant areas of activation from the ANCOVA analysis with raw, rather than Rankit transformed, aggression level scores as the covariate. Activations are from whole brain analyses significant at p<0.001, corrected for multiple comparisons (significant at p<0.05).

| Region | BA | Voxels | X | Y | Z |
| --- | --- | --- | --- | --- | --- |
| Aggression level-by-Direction | | | | | |
| R. MTG/STG | 41 | 34 | 41 | -43 | 8 |
| R. IPL | 40 | 27 | 26 | -34 | 62 |
| R. Uncus | 20 | 50 | 23 | -13 | -22 |
| L. Culmen |  | 42 | -19 | -46 | -19 |
| Aggression level-by-Direction-by-Emotion | | | | | |
| R. Precuneus/PCC | 7 | 8 | 2 | -58 | 32 |

Key to Supplemental Table 4: MTG: Middle temporal gyrus; STG: Superior temporal gyrus; IPL: Inferior parietal lobule; PCC: Posterior cingulate cortex.

**Supplemental Material 4: Legends for Supplemental Figures**

*Supplemental Figure 1:* An exemplar trial of an adapted version of the Looming task (Coker-Appiah et al., 2013). Images were rapidly presented in a series of sixteen 50-ms frames of increasing or decreasing size in the center of the screen to create the effect of looming (i.e., increasing in size over time) or receding (i.e., decreasing in size over time; total stimulus duration: 800 ms). Stimulus presentations were followed by a fixation point, which was on screen for a jittered duration of 1250-4250 ms.

*Supplemental Figure 2:* The region of right amygdala showing a main effect of direction; (a) Parameter estimates for looming and receding trials for right amygdala; (b) Negative association of differential (looming-receding) BOLD response and Aggression Level; (c) Negative association of differential (looming-receding) BOLD response and total RPQ score; and (d) Negative association of differential (looming-receding) BOLD response and RPQ proactive aggression score (the correlation with RPQ reactive aggression score was non-significant (r=-0.178, p=0.090)).
