# Supplementary figures and images for: Reduced neural responsiveness to looming stimuli is associated with increased aggression
Source: Soc Cogn Affect Neurosci. 2021 May 7;16(10):1091–9. doi: 10.1093/scan/nsab058 (PMC8483278; doi:10.1093/scan/nsab058)

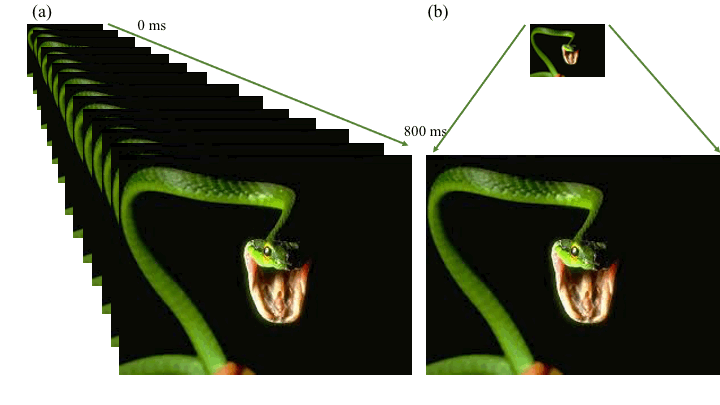

Supplement: nsab058_Supp [file nsab058_supp.zip › Supplemental_Figure_1.gif]

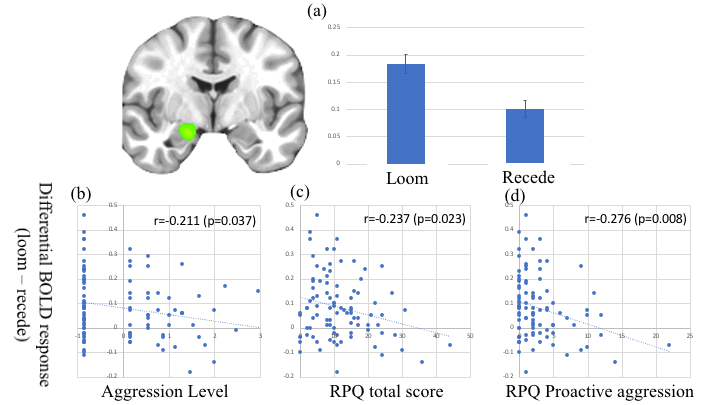

Supplement: nsab058_Supp [file nsab058_supp.zip › Supplemental_Figure_2.gif]
